# Waterpipe smoking: not necessarily less hazardous than cigarette smoking: Possible consequences for (cardiovascular) disease

**DOI:** 10.1007/s12471-013-0501-0

**Published:** 2013-12-05

**Authors:** J. B. Jukema, D. E. Bagnasco, R. A. Jukema

**Affiliations:** 1 Medical Faculty, Free University, Amsterdam, the Netherlands; 2 Visser ‘t Hooft Lyceum, Leiden, the Netherlands

**Keywords:** Waterpipe, Smoking, Health effects, Cardiovascular, Awareness

## Abstract

**Context:**

Cigarette smoking has declined over the last years in modern countries. On the contrary, waterpipe smoking has increased, especially among young people visiting waterpipe bars. Unfortunately, most waterpipe smokers seem to know little about the possible cardiovascular and other health consequences of waterpipe smoking.

**Objective:**

To describe by narrative literature review the known adverse consequences for the human body caused by smoking the waterpipe compared with the consequences of smoking normal cigarettes. Also, to get a picture of public awareness of these consequences as deducted from the literature and a small new survey in the Netherlands.

**Results/Conclusions:**

Tobacco smoking is associated with serious adverse (cardiovascular) health effects, and there is no evidence that these effects are less serious if a waterpipe is used. The increasing use together with the limited amount of awareness and attention for the possible health consequences of smoking the waterpipe is worrisome. Especially considering the increasing acceptance and use of the waterpipe among the youth. Therefore we recommend more systematic research into the possible health hazards of waterpipe smoking. In the meantime education campaigns and materials are needed to raise public awareness on the possible health risks of waterpipe use.

## Introduction and Methods

Cigarette smoking has declined over the last years in modern countries [1,2], in contrast to smoking the waterpipe. The waterpipe originates from Indian/Arab countries. It is becoming rapidly more popular in Western society, including the Netherlands, especially among young people in waterpipe bars. However, neither waterpipe smokers nor health professionals are well informed about the possible health consequences. We aimed to perform a systematic weighted review to answer the main question, i.e. what are the possible consequences for the human body of smoking the waterpipe in comparison with smoking normal cigarettes, and what is the public awareness of these consequences? However, too few data were available for such a systematic weighted review/meta-analysis. Therefore we instead performed a narrative literature search using the search words in PubMed/Medline/Embase: waterpipe, smoking, hookah, shisha, nar(g)hile, health, public awareness and survey. We checked the retrieved articles for appropriateness and for other relevant references. One of the most important reports we retrieved, used and crosschecked was a public-access manuscript from the National Institutes of Health (NIH) [3]. Also internet was checked for these terms because many (commercial) messages and activities about waterpipe smoking appear on or are reported about on the internet. Since it became apparent that very little data were available on waterpipe smoking behaviour in Western Europe/the Netherlands, we also performed a small orientating survey in the Netherlands (around the city of Leiden). We used the online survey site surveymonkey.com for this purpose.

## Backgrounds, History, Trends and Analysis of the current Dutch Situation

Nearly a millennium ago the waterpipe already emerged in the North Western provinces of India along the border of Pakistan. Those waterpipes were constructed from the base of a coconut with an attached head and tube. The original purpose of the waterpipe was to smoke opium and hashish. From India the waterpipe spread to Iran and the rest of the Arab world. Because use of the waterpipe spread over a large area with different languages, the waterpipe obtained quite a few different names. Commonly heard terms for the waterpipe are: hookah, shisha, nargile, arghile and hubble bubble. In Turkey the waterpipe also became extremely popular in the upper class, changing its design. The waterpipe gained size and complexity and became similar to the waterpipe that is used today. The elite developed an etiquette for waterpipe use. In Turkey the first waterpipe bars arose about two to three centuries ago. In the 19th century cigarettes became widely available so men transferred from the waterpipe to cigarettes because it was a more mobile form of smoking. Women kept smoking the waterpipe because they had to stay inside and used it as a pastime. Most hookah smoking countries serve Naklia shisha (also known as maassel). Naklia shisha is a combination of foreign tobaccos, honey molasses and dried fruit. The smoke is filtered through the cold water to make the smoke cool and soothing. Because of the original purpose (opium and hashish) the waterpipe had a negative stigma but nowadays the popularity of the waterpipe is rapidly increasing and seems to get a lot of positive attention and appraisal. In Western Europe, including the Netherlands, the waterpipe was only recently introduced.

‘Cigarettes are for nervous people, competitive people, people on the run […] When you smoke a narghile (waterpipe), you have time to think. It teaches you patience and tolerance, and gives you an appreciation of good company. Narghile smokers have a much more balanced approach to life than cigarette smokers.’ (Ismet Ertep, 71 years Turkey)[4,5]

## Working of the Waterpipe

**Fig. 1:**
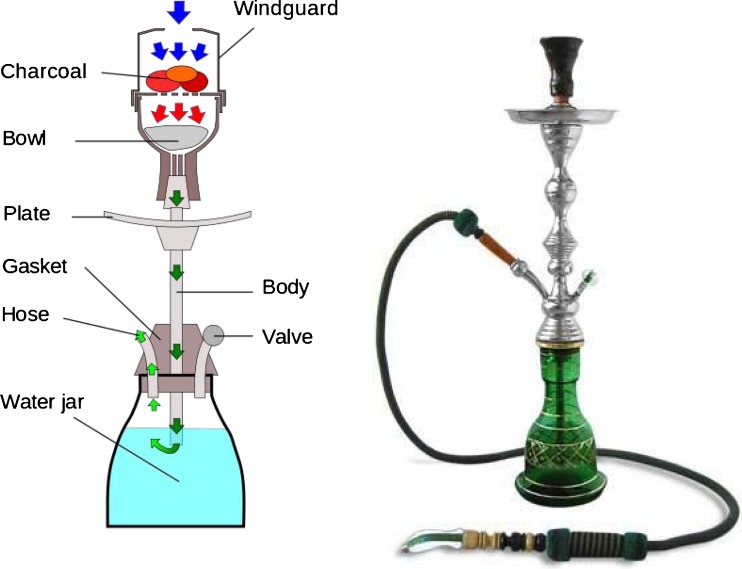
The waterpipe (schematic and picture) and its essential parts (explanation see text, source Wikipedia)

The waterpipe may look complex but it is straightforward to use. In order to get some background knowledge of the waterpipe, its parts and their function are briefly discussed here (see also Fig. 1). The lüle is a bowl where the tobacco is placed. The coals which burn the tobacco are placed on this bowl. The coals and the tobacco are usually separated by aluminium foil.

If a user of the waterpipe sucks up the air from the valve the air passes through the bowl with tobacco. The air is heated up by the burning coals on top of the bowl. So the air obtains its heat in the beginning of the process. Thereafter the smoke goes down the body through a hollow tube to the water jar. In the water the smoke is cooled down and gains moisture. Therefore waterpipe smoke is much easier to inhale deep into the lungs compared with cigarettes. The jar can not only be filled with water but also with other liquids, such as drinks with alcohol, fruit juice or milk. After passing the water jar, the smoke proceeds through the hose into the mouth and lungs of the smoker.

## Possible Myths about the Waterpipe

Waterpipe use is relatively new in the West, so there are many possible myths surrounding the practice. The most important myth is supposedly that the use of the waterpipe is not as bad for your health as smoking cigarettes. In a US survey in 2007, 58 % of waterpipe smokers believed that waterpipe smoking was less harmful than cigarettes, 31 % believed it to be more harmful and 11 % considered it probably equally harmful [6]. Several recent US college-based studies show that the majority of waterpipe tobacco smokers perceive this tobacco use method as being less harmful [6,7]. Research has proven that, contrary to what people think, waterpipe tobacco smoke can be as harmful or even more harmful than cigarette smoke. Waterpipe tobacco smoke contains 6.5 times more CO, 1.7 times more nicotine and 46 times more tar (see later in this article). Another reason why people might consider a ‘reduced’ harm may be related to the predominantly intermittent use of waterpipe smoking. [8]. The fact that a single episode of waterpipe smoking involves a bigger quantity of smoke than a single cigarette suggests that an intermittent pattern of waterpipe tobacco smoking may involve equal levels of smoke toxicant exposure. Many users, however, believe that waterpipe smoke is far less harmful than cigarette smoke because the smoke passes through water which they presume acts as a filter. Unfortunately, the water only acts as a cooling agent, not as a filter for nicotine, tar or carcinogens. And, the cooling process forces the smoker to inhale much deeper. Deeper inhalation causes the smoke to penetrate deeper into the lungs, which may cause additional health effects. A second possible myth is the fact that cigarette smoking is more addictive than waterpipe tobacco smoking.[8] Of beginning waterpipe users, 90 % believe cigarette smoking is more addictive, according to the American Lung Association. This assumption is not correct. Waterpipe tobacco and smoke contain the same addictive drug found in cigarettes, nicotine. Many smokers may suggest they do not inhale the smoke. However, even if you only take the smoke into your mouth and you do not actually inhale, your body still absorbs the nicotine through the lining of your mouth. Another possible myth is that only regular waterpipe smoking causes diseases. This may also be false. Waterpipe users often share mouthpieces, so even incidental waterpipe users are exposed to risks of getting a cold, herpes, oral bacterial infections and even tuberculosis.

## (Waterpipe)Tobacco Trends and Policies of the 21^st^ Century

All tobacco together caused an estimated 100 million deaths in the 20th century. According to the World Health Organisation it will cause up to one billion deaths in the 21^st^ century if the current trend continues. Currently the waterpipe is getting more popular for some major reasons. These include: introduction of a flavoured tobacco mix, the mushrooming of hookah establishments, and aggressive marketing and media hype about this new trend. One of the characteristics of the waterpipe that attracts a lot of people is the aromatic smell [9–11]. This aromatic smell is caused by the slow heating with charcoal of a tobacco that consists for about 30 % of crude cut tobacco and is fermented with about 70 % of honey molasses (syrup) and the pulp of different fruits. This new kind of tobacco called ‘maassel’ was introduced by some Egyptian tobacco companies in the early 1990s. Another reason is the emerging of hookah bars, cafés and restaurants. In 2004 the Smokeshop magazine reported that 200 to 300 hookah bars have been opened in the United States since 1999. These cafés were ‘often near college campuses’ [12,13]. Waterpipe hookah bars, cafes, and restaurants lure customers by advertising in college/university, local newspapers and radio stations popular among young people. They emphasise exotic aspects of Middle Eastern culture in their décor, furnishings, music, and displays of a variety of colourful, finely crafted hookahs [14]. Further, the increasing popularity can be due to the aggressive marketing on hookahs, hookah accessories and maassel of multiple enterprises that have sprung up in the US and the Middle East. To attract customers, these businesses offer a variety of hookahs for sale, e.g. Egyptian Hookahs, Sheik Hookahs, Rotating Hookahs, and Modern Hookahs or give these products exotic names such as ‘Sheherazade,’ ‘Syrian Queen,’ and ‘Queen Nefertiti.’ Some websites promote hookah use as chic and elegant (hookahculture.com), as part of a unique lifestyle (insidehookah.com) or religious worship (sacrednarghile.com) [14]. The last big contributing factor is the amount of media attention. It can almost be called a media hype. The waterpipe has been given quite a lot of attention, more positive than negative. Some newspapers warn about its potential health risks but most depict it as a new, trendy and safe way of socialising for young people [15]. Waterpipe tobacco smoking is often associated with Southwest Asia and North Africa. A recent study in Southwest Asia and the United States suggests that children from an Asian background start smoking at a relatively young age. For example, in a survey of 2443 Lebanese students (11 to 17+ years old; *M* = 15) from public and private secondary schools in greater Beirut, 64.9 % reported that they had tried waterpipe at some point in their life and 25.6 % reported use in the last past 30 days [16]. Also, among 388 Israeli schoolchildren aged 12-18 years, 41 % reported current waterpipe tobacco smoking, and 22 % reported that they used a waterpipe to smoke tobacco every weekend [17]. These data are also about juvenile smoking. All of the data addressing young people are important especially because, at least for cigarette smoking, earlier initiation is associated with longer duration of smoking and increased risk of nicotine dependence and deleterious health effects [18]. Moreover, waterpipe tobacco smoking may be introducing tobacco to an otherwise tobacco-naive group of adolescents and young adults. In Pittsburgh, 35.4 % of university students who use a waterpipe had never smoked a cigarette. There are several reports stating that the United States and probably also Europa are on the brink of a waterpipe epidemic amongst its college-age population. Most of the reported increasing amounts of waterpipe use (at least 33 states reported waterpipe use) come from cities with a large university [19–26]. Hookahs have become commonplace at fraternity parties at these universities’ [27]. Young waterpipe users may also be attracted to the aromatic smell of the waterpipe smoke [10,28]. Arab Americans describe the waterpipe as cool and as an opportunity for social interaction [29]. The keyword that comes back in almost every study on the popularity of the waterpipe is socialising. One study resulted in the statistic that 79 % of the 201 tobacco smokers did so, at least partially, because of the social interaction [7]. A waterpipe is meant to be smoked socially unlike normal cigarettes that can only be smoked outside or in smoking rooms and are thus destroying the social fun. Another surprising outcome of surveys is that girls are more comfortable with a waterpipe than they are with a cigarette.

A worrisome fact is that many waterpipe tobacco smokers are otherwise tobacco naive. Active individuals who enjoy the effects of smoking tobacco with a waterpipe may turn to cigarettes for a more convenient and mobile smoking method. [30]. Such a prospect is plausible given the fact that waterpipe tobacco smoking is time-consuming and largely site specific. Evidence from a cross-sectional study of Arab American adolescents shows that the odds of experimenting with cigarettes were 8 times greater for those who have ever smoked tobacco using a waterpipe [31,32]. Another study among young adult US military recruits shows that waterpipe smokers are more likely to start smoking cigarettes in the following year than non-waterpipe smokers [32]. Furthermore, there is less to no evidence that waterpipe tobacco smoking is related to marijuana use. A survey under 201 US waterpipe tobacco smokers reported that 64.2 % had not used marijuana in the past 30 days and only 10.4 % reported that they had smoked tobacco and marijuana from the same waterpipe [7].

## Survey in the Netherlands

Since data on waterpipe habits and perception were mainly available for Asian and US regions and there was little information for Europe, we performed a small additional survey in the (university) city of Leiden, the Netherlands. From our surroundings, we gathered 133 responses (response rate 84 %) at surveymonkey.com [33]. The respondents were male (53 %) and female (47 %). The majority of the respondents had a (pre-)university background (78 %). Most respondents (62 %) were 16 to 18 years. Another large group of respondents (14 %) was aged 21-34 years.

**Fig. 2:**
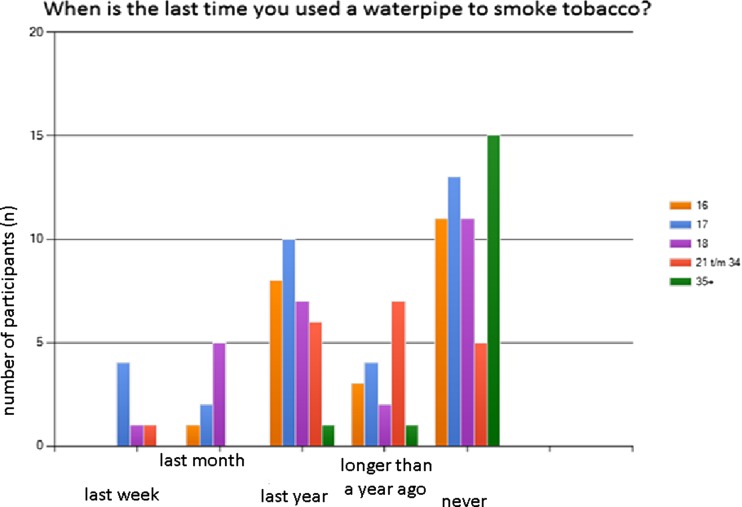
When is the last time you used a waterpipe to smoke tobacco, according to age category (years)

One of the interesting results of our survey is that the vast majority (73 %) never smoked cigarettes but more than half of the respondents (52 %) had ever smoked a waterpipe. Even 38 % smoked a waterpipe last year! The two most important possible myths about waterpipe smoking were clearly visible, especially the myth about the possible (non-)addictiveness of the waterpipe. Around 95 % of respondents do not think waterpipe smoking is addictive and certainly not to the same extent as cigarette smoking. We also recognised the other possible myth about little health consequences of waterpipe smoking. A little more than one-third of the questioned persons have the idea that smoking the waterpipe could be more harmful than smoking cigarettes. This indicates that about two-thirds of the respondents are in ignorance about the possible deleterious effects on health of smoking the waterpipe relative to smoking cigarettes. In order to get a deeper understanding of the use of the waterpipe between different age groups, we selected 5 groups with in total 118 respondents. Figure 2 demonstrates that younger age groups (teenagers) have a relatively high usage of the waterpipe. All our results for the Netherlands are in concordance with the previously discussed non-European surveys. The results indicate that waterpipe popularity is clearly growing, especially amongst adolescents that otherwise are non (cigarette) smokers. Also it indicates that this group is largely unaware of the possible serious health hazards of waterpipe smoking.

## Detailed Description of Toxicological and Health Issues

### Substances Present in the Water Pipe

The literature describes proper scientific experiments that have been conducted to uncover the true health risk of the waterpipe, also compared with cigarettes. For some tobacco smoke toxicants (e.g. nicotine, carbon monoxide (CO)), the smoke content and user toxicant exposure associated with waterpipes is at least comparable to that of cigarettes [3,34–36]. When waterpipe tobacco smoke is generated by a machine that is programmed to imitate the puff parameters of actual waterpipe users, substantial amounts of nicotine, CO, and tar can be measured [37–39]. Compared with a machine programmed to smoke cigarettes, the levels of CO and tar produced by a single waterpipe use are substantially greater [40]. However, there is a difference between the quantities of smoke inhaled. Data collected from actual waterpipe tobacco smokers in natural settings show that a waterpipe use episode typically involves almost 200 puffs, with an average puff volume exceeding 500 ml [39,40]. Thus, a single waterpipe use episode involves inhalation of approximately 90,000 ml of smoke [39,40]. A cigarette inhalation involves approximately 500–600 ml of smoke (i.e. 10–13 puffs of about 50 ml, on average [41,42]), Although these detailed puff topography data are based on waterpipe tobacco smokers in Lebanon, the duration of waterpipe use episodes has been explored (via self-report) in surveys of US waterpipe tobacco smokers. Of those smokers, 44 % report episodes of 60 min or longer [7]. Relative to a single cigarette (about 500 ml of smoke), a single waterpipe episode is associated with 1.7 times the nicotine, 6.5 times the CO, and 46.4 times the tar [42]. Research has proven that waterpipe tobacco smokers are exposed to these and several other smoke toxicants. The most important one is the psychoactive and dependence producing drug nicotine. Another harmful toxicant found is carbon monoxide (CO), which reduces the blood’s ability to carry oxygen. In a recently published study, scientists proved that waterpipe tobacco smoking led to a mean increase in expired air CO of over 30 ppm (part per million). This is about 5 times the amount expected from a single cigarette. Although more research is needed, preliminary evidence supports that waterpipe users are exposed to other toxicants in waterpipe smoke. Some examples are: lung carcinogens [38,43] and heavy metals [37,44].

Research has shown that waterpipe users are subjected to other toxicants also found in cigarettes. For example machine-generated waterpipe smoke contains disturbing levels of volatile aldehydes such as formaldehyde, acetaldehyde and acrolein, all compounds that can be found in cigarette smoke [45]. The isotope ^210^Po, which is a member of the uranium decay series and detected in tobacco smoke, is another worrisome toxicant. It is a radioactive compound which can deliver serious radiation doses and cause radiotoxic effects to humans [46,47]. In comparison with cigarette smoke, the concentration of the ^210^Po isotope is lower in waterpipe smoke but still high[48,49].

Smoke machine experiments have also shown that waterpipe smoke delivers toxic and carcinogenic compounds such as arsenic, beryllium, chromium, cobalt, lead and nickel. These toxicant metals originate from the coal used in the waterpipe. In Table 1 possible toxicants and health hazards of waterpipe smoke and smoking are presented. However, waterpipe users are not the only ones who are exposed to the waterpipe-associated toxicants; nearby non-smokers may also come in contact with the harmful compounds. Other studies show that smoke from a waterpipe contains high doses of small particulate matter (PM _2_._5_) [50,51] which plays an important role in damaging the cardio and respiratory systems [52,53]. By smoking waterpipe tobacco these particles are emitted in the air, reaching levels comparable to cigarette smoking.

**Table 1:** Possible health hazards of waterpipe smoking (from the smoke, the coal or the pipe system)

	Health risks	Reference
Hazardous elements in smoke		
CO carbon monoxide	• Subtle cardiovascular disorders	[34,38,64,82]
	• Neurobehavioral effects	
Tar	• Tumourgenicity	[1,10,38]
	• Mutagenicity	
Nicotine	• Nicotine intoxication	[36,38,64,83]
	• Accelerated atherosclerosis	
	• Stroke	
	• Hypertension	
	• Delayed wound healing	
	• Reproductive and perinatal disorders	
Volatile carcinogenic nitrosamines	• Bronchogenic carcinoma	[66–73]
	• Lung cancer	
	• Oral cancer	
	• Bladder cancer	
Volatile aldehydes	• Cancer	[45]
Polonium 210	• Bronchial cancer	[46,47]
Hazardous elements in coal		
Arsenic	• Hypertension	[1,38,84]
	• Diabetes mellitus	
	• Lung cancer	
	• Bladder cancer	
	• Keratosis	
	• Hyperpigmentation	
Nickel	• Allergic reactions	[1,85]
	• Hypersensitivity	
	• Headaches	
	• Pulmonary cancers	
	• Hepatic toxicities	
	• Renal toxicities	
	• Asthma	
	• Bronchitis	
	• Rhinitis	
	• Sinusitis	
	• Pneumoconiosis	
Chromium	• Lung cancer	[1,86]
Cobalt	• Allergic dermatitis	[1,87]
	• Rhinitis	
	• Asthma	
	• Intense alveolitis	
	• Pulmonary fibrosis	
Lead	• Atherosclerosis	[1,88–90]
	• Neurotoxic effects	
	• Male infertility	
Hazardous elements in pipe		
Bacteria:		
• Mycobacterium tuberculosis	• Tuberculosis	[62,63]
Viruses:		
• Herpes	• Irritated skin	[62,63]
	• Blisters	
	• Skin ulcers	
	• Fever	
• Hepatitis	• Liver damage	[62,63]

### Health Consequences for the Human Body

In comparison with cigarettes, not much research has been done on the effects of waterpipe tobacco smoking on the human body, although the history of the waterpipe is much longer than that of the cigarette. This lack of research might be the result of a lack of adequate resources in the regions where the waterpipe originates. Since waterpipe tobacco smoke harbours many different toxicants and carcinogenic compounds, waterpipe use may result in serious health consequences. Some health effects may be short term while others may be long term.

#### Short-Term Health Consequences

Smoke constituent delivery of the waterpipe can be tested in controlled conditions. Substances as nicotine and CO, as well as cardiovascular and other effects, can be assessed. As indicated, research discovered that expired air CO, plasma nicotine, and heart rate are significantly elevated after 45 min of waterpipe smoking [54,55]. Another, more precise, cross-study concludes that 45 min of waterpipe smoking about doubles the concentration of CO in the blood and triples nicotine exposure compared with one normal cigarette [56]. Also, there is a clear effect on the heart, because haemoglobin has an affinity for carbon monoxide that is 240 times as high as for oxygen. The lack of oxygen induces a (temporal) rise in heart frequency and an increase in heart workload. In the research about haemoglobin just described, [55] cigarettes were compared with a waterpipe. This is of course a dangerous comparison because a cigarette just lasts about five minutes while a waterpipe session will take about 45 min. Still, this study is consistent with a field study in which samples from cigarette smokers (*n* = 601) and waterpipe users (*n* = 975) were analysed for carboxyhaemoglobin (COHb): relative to smokers’ average COHb concentration (6.47 %), waterpipe users’ scored significantly higher (10.06 %) *p* < 0.001 [57,58]. As might be expected, the number of waterpipe uses per day was highly correlated (*r* = 0.84, *p* < 0.001) with COHb [57]. Another risk of waterpipe and cigarette is addiction, or rather nicotine dependence. It is commonly known that cigarette smokers have a hard time stopping, in spite of the known potential health risks and financial costs. From research published we can conclude that the amount of nicotine a waterpipe user engulfs is higher, or at least equal to the amount of cigarette smoke that gets in his body. So, just like cigarette smokers, waterpipe smokers are at risk of developing a physical dependence to this psychomotor stimulant [55,59]. No studies have characterised a waterpipe withdrawal syndrome yet. However, waterpipe tobacco smokers have certain characteristics that point towards an addiction; continued use despite potential health risks, financial cost and difficulty to quit [60]. The same study suggests a possible definition of a waterpipe addiction; it may be the transition from smoking as primarily a social phenomenon (that is, while relaxing with family or friends, often in restaurants or cafés) to a more solitary experience (that is, alone, at home). In some of the countries where the waterpipe originates, such as India, it is impolite not to offer a waterpipe to the guest [61]. In Beirut, Lebanon, 89.8 % of the questioned waterpipe smokers share the waterpipe with friends [27]. So the waterpipe is often shared with many fellow smokers. This practice can spread tuberculosis [62,63] and viruses such as herpes and hepatitis. To prevent this spread, the use of disposable mouthpieces, which is currently becoming more popular, may be helpful though bacteria and viruses will still be able to persist in other parts of the waterpipe than the mouthpiece, like the hose.

#### Long-Term Health Effects

There is considerable evidence linking waterpipe use to disease. Unfortunately not all research is reliable since a lot of the waterpipe users smoke cigarettes and engage in other kinds of behaviours which increase the risk of cancer. A recent well-performed large study among 50,000 residents of Golestan clearly showed a significant association between coronary heart disease and heavy waterpipe smoking, which is in concordance with earlier smaller studies [64,65]. Waterpipe use likely increases the risk of bronchogenic carcinoma [66], as well as lung [67–69], oral [70], and bladder [71,72] cancers. This is not surprising because, like cigarette tobacco, the tobacco used in the waterpipe contains high amounts of volatile carcinogenic nitrosamines. [73] In a study comparing 35 healthy waterpipe users with 35 non-exposed controls, waterpipe use increased the number of chromosomal mutations and sister chromatid exchanges. Compared with controls, waterpipe users were also subjected to an alleviated mitotic index. There are other long-term diseases with which waterpipe use is linked. Several studies have examined its pulmonary effects [62,63,74–79]. Two studies determined the function of the lungs of waterpipe users compared with cigarette smokers and non-smokers. Although there was a difference in magnitude of effect of waterpipe use on lung function between the two studies, they both demonstrated a difference in average forced vital capacity and forced expiratory volume in 1 s of waterpipe users compared with non-users [75,80]. Additionally, relative to cigarette smokers, the airflow at 25-75 % vital capacity was significantly lower in waterpipe users [78]. Also peak flow rate was less than 200 l/min in 37 % of waterpipe users, compared with 3.8 % of cigarette smokers [62].

#### Impact of Foetal Exposure

Carbon monoxide during pregnancy can harm the foetus, and is often thought to be the reason for the low birth weight observed in babies born to smoking mothers (foetal tobacco syndrome). Sadly there are no studies regarding the specific extra harmful effects of the heavy metals. Women who smoke waterpipe tobacco during their pregnancy put their babies at risk. The probability that babies are born with low weight and respiratory distress increases drastically due to use of the waterpipe [81].

#### Conclusion and Recommendations

Waterpipes have been used for centuries to smoke tobacco, but over the past few decades this device has experienced an unprecedented rise in popularity in the US, Middle East and Europe, including the Netherlands. A lot of waterpipe users see themselves as non-smokers if they do not smoke cigarettes as well. Several surveys including our own show this rise in popularity, especially among adolescents, who have encountered the waterpipe more often than the adult generations. Several factors are responsible for this increase such as the introduction of flavoured tobacco, the rise in the number of waterpipe establishments, aggressive marketing tactics by businesses and newspaper articles and other kinds of media that have improved the image of the waterpipe. Due to these factors, many users consider waterpipe smoking (almost) harmless and not real smoking. This seems in contrast with the actual figures from the literature where it is shown that waterpipe smoke contains significantly more carbon monoxide, nicotine and far more tar than cigarette smoke. Moreover, this waterpipe smoke may also contain high doses of heavy metals coming from the used coal. All these toxicants present in the waterpipe smoke may have serious consequences for the human body, both in the long and the short term, such as cardiovascular disease, reduced pulmonary function, several types of cancers and foetal exposure. Unlike cigarette smokers, waterpipe smokers have the additional risk of obtaining diseases as tuberculosis and viruses, due to direct or indirect waterpipe sharing.

Therefore, we recommend more systematic research into the possible health hazards of waterpipe smoking. In the meantime, education campaigns and materials are needed to raise public awareness on the possible health risks of waterpipe use, in this way dispelling the myths that it is by definition safe to use.
